# Enhanced susceptibility of pediatric airway epithelium to respiratory syncytial virus infection

**DOI:** 10.1172/JCI185689

**Published:** 2024-11-01

**Authors:** Raymond J. Pickles, Gang Chen, Scott H. Randell

**Affiliations:** 1 Marsico Lung Institute,; 2 Department of Microbiology and Immunology,; 3 Department of Pediatrics,; 4 Department of Cell Biology and Physiology, The University of North Carolina at Chapel Hill, Chapel Hill, North Carolina, USA.

## Abstract

Immature innate and adaptive immunity and vulnerability of narrower airways to obstruction increase the susceptibility of infants to severe respiratory syncytial virus (RSV) disease. In this issue of the *JCI*, Zhao et al. illustrated greater intrinsic susceptibility of pediatric versus adult airway epithelial cells to RSV-induced cytopathology. Using precision cut lung slices (PCLS) and air-liquid interface (ALI) airway epithelial cell cultures, the authors showed that impaired STAT3 activation in RSV-infected pediatric multiciliated cells increased cell apoptosis and viral shedding, which enhanced the spread of infection. Bolstering STAT3 activation and treatment of neonatal mice with apoptosis inhibitors suppressed virus spread, suggesting that enhancing STAT3 activation may provide therapeutic benefit.

## RSV infections across the human life span

Almost all children experience at least one respiratory syncytial virus (RSV) infection by 3 years of age (1, 2). RSV infections are more severe in infants under 6 months of age, with neonates under age one month particularly at risk for hospitalization (3). Although underlying pulmonary disease (e.g., cystic fibrosis) and T cell immunodeficiencies (e.g., DiGeorge’s syndrome) increase susceptibility to more severe infections, 80% of infants with severe RSV infection are otherwise healthy (3). Polymorphisms in innate and adaptive immune genes (e.g., encoding TLR4, surfactant proteins, HLA proteins, and MUC4) and environmental exposures such as cigarette or wood smoke have been linked to increased RSV severity (4). RSV immunity wanes and reinfection throughout childhood is common, often with the same virus subtype. However, lung disease severity typically decreases up to and through middle age, largely attributed to improved immunity after cumulative RSV exposures. Susceptibility to severe RSV-related pulmonary disease returns in individuals over 75, with mortality rates similar to influenza viruses (5, 6). Recently, new RSV vaccines and more potent antibody approaches have been approved to lessen the impact of infection in infant and older adult populations.

## Cellular models for investigating RSV infection

Early pioneering respiratory virus studies employed ex vivo tissue explants containing virus-trophic differentiated cells (7). Air-liquid interface (ALI) respiratory tract epithelial cell in vitro cultures now routinely replicate organotypic cellular differentiation, and such cultures reveal important consequences of RSV infection not manifested in commonly used cell lines. Previous studies demonstrated robust RSV infection and epithelial cytopathology in pediatric (8) and adult (9) ALI cultures. Extensive epithelial cell expansion and creation of ALI cultures from minimally invasive endotracheal tube aspirates is now possible (10), and precision cut lung slices (PCLS), originally employed to study bronchoconstriction, are now being more widely used to study infection and fibrosis (11). As reported in this issue of the *JCI*, Zhao et al. are the first to perform side-by-side comparisons of RSV infection of pediatric- versus adult-derived well-differentiated airway epithelial cells (12). They used PCLS and ALI cultures to examine airway epithelial responses to RSV that may explain, at the epithelial cell level, why infants suffer worse RSV infections than adults. Despite equal virus binding and intracellular replication, the authors show more extensive shedding and apoptosis in RSV-infected pediatric-derived multiciliated cells than in equivalent cultures of adult-derived cells and that greater epithelial cytopathology enhanced the spread of infection. Decreased susceptibility of adult-derived cultures to shedding and apoptosis of RSV-infected multiciliated cells is an intriguing finding suggesting that intrinsic differential airway epithelial cell properties vary with age, providing another potential reason why infants suffer worse RSV infections than adults.

Ciliated cell shedding due to RSV infection and its role in facilitating virus spread in cell cultures was demonstrated previously (9, 13) and was confirmed in Zhao et al. (12). However, it is important to note that spread of RSV infection in vitro is confined within the enclosed culture system, constraining removal of progeny virus and/or shed epithelial cells. Shed progeny virions and epithelial cells are transported across the apical surface of airway cultures but are not mechanically cleared from the culture, increasing virus retention time and leading to more infectious spread. In contrast, progeny virions and multiciliated cells shed in vivo can be removed from infection sites, at least initially, by unidirectional mucociliary transport and cough clearance of airway secretions. Studies in hamsters demonstrated that epithelial cell shedding in response to respiratory virus infection reduced virus loads, suggesting that cell shedding may provide a protective mechanism by purging the virus (9). Whether apoptotic cell shedding facilitates protection versus spread will be determined by virus susceptibility of remaining airway cells, efficiency of airway luminal debris clearance, and immunological memory and responses, all of which are factors likely to change with age.

Important technical considerations related to the cell culture models include the primary epithelial cell anatomical source. In the Zhao study (12), cells were obtained from endotracheal tube aspirates. Whether the obtained epithelial cells were predominantly tracheal or from more distal airways and transported to the endotracheal tube via mucociliary clearance, and if the cell populations were systematically different in neonates and adults, is not clear. Small numbers of stem and progenitor cells potentially at different initial developmental stages underwent multiple population doublings under specific expansion and differentiation conditions in culture (10). Bulk mRNA sequencing of ALI cultures in Zhao et al. (12) and another study using larger initial cell numbers (14) revealed many age-dependent differentially expressed airway epithelial genes. While cells from young and adult donors appeared similar morphologically and were equally susceptible to SARS-CoV-2 in Zhao et al. (12), another study noted differences in coronavirus growth from respiratory cells derived from young versus older patients (15). No in vitro studies fully replicate in vivo conditions that include contributions of stromal and immune system cells likely affecting age-dependent susceptibility. The PCLS model used in Zhao et al. (12) retains some but not all (e.g., circulating cells) of the contributing cellular compartments, and understanding age related differences in other cell types and in the integrated biological system in vivo is an area for future investigation.

## STAT3 activation prevents apoptosis and anoikis

Using gene expression analysis of pediatric- and adult-derived cultures, Zhao and authors identified hyporesponsive STAT3 signaling as a potential reason why pediatric cells were more susceptible to RSV-induced cell shedding and apoptosis (12). STAT3 activation is a well-established molecular mechanism to suppress cellular apoptosis, and STAT3 activation is upregulated in many immortalized cancer cell lines (16). In this context, RSV has been previously shown to activate STAT3 (17), although modulation of STAT3 by other respiratory viruses is variable and complex (18). Using genetic and pharmacologic methodologies to modulate STAT3 activation in the culture models, the authors convincingly show that RSV activation of STAT3 was more robust in adult-derived cells than in pediatric-derived cells and that modulation of STAT3 activation could substantially affect multiciliated cell shedding and apoptosis in response to RSV.

**Figure 1:**
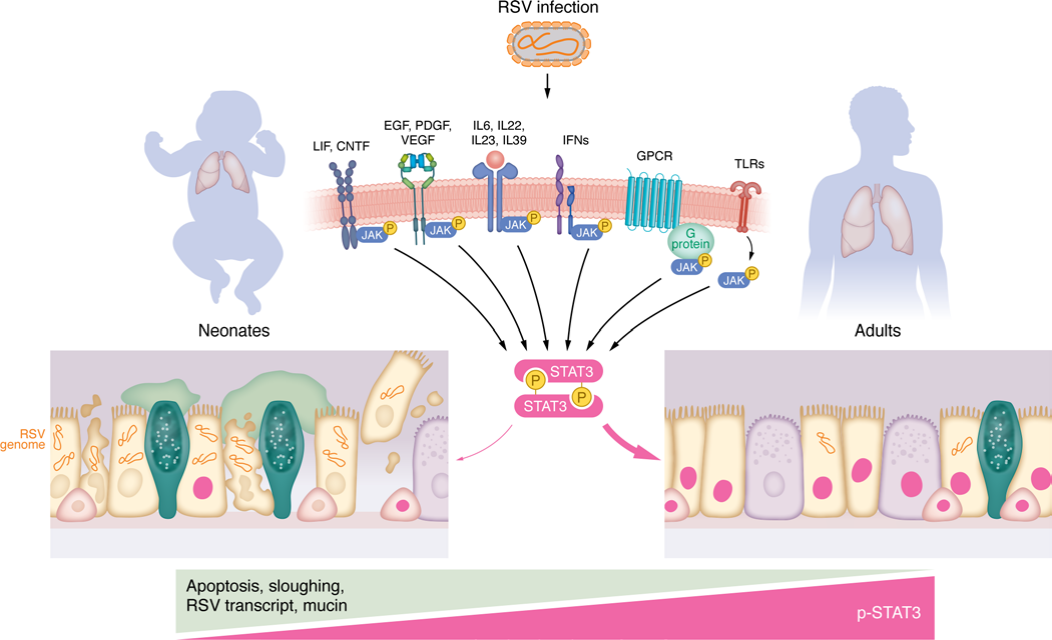
STAT3 hypoactivation in neonatal/pediatric airway epithelial cells increases spread of RSV infection. RSV infection of airway epithelial cells from infant and adult lung tissue triggers STAT3 phosphorylation, causing STAT3 activation and nuclear translocation, particularly in multiciliated and basal cells. This process can be mediated by Janus kinase (JAK) following the binding of various ligands and agonists to their respective receptor complexes following infection. In pediatric airway epithelial cells, reduced STAT3 activation in response to RSV results in increased apoptosis, cell shedding, and mucin production compared with adult airway cells. Safely managing epithelial STAT3 activation may be a viable therapeutic strategy to control RSV infection in neonates.

Apoptosis is a host defense mechanism to remove compromised cells and, as noted above, likely serves as a host defense mechanism to reduce viral load. In adherent epithelial cells, cell detachment–induced apoptosis is known as anoikis, and avoidance of anoikis is a critical event for carcinoma metastasis (19). Indeed, many viruses, including RSV, have evolved genes to suppress host cell apoptosis and enhance virus growth (20). However, some viruses exploit the apoptotic cellular response to facilitate spread (21). Data presented in Zhao et al. (12) indicate that hyporesponsive STAT3 activation in pediatric cells during RSV infection failed to sufficiently suppress apoptosis, enhancing spread of infection. The proximal cause of STAT3 hyporesponsiveness in pediatric-derived cultures or, alternatively, STAT3 hyperresponsiveness in adult-derived cultures, was not fully explored in this study (12). STAT3 can be activated by multiple secreted factors that activate IL6R, EGFR, TLRs, and specific IFNRs (Figure 1) (22). Determining the status of these important receptors and their signaling pathways in infant- versus adult-derived cultures would help define the mechanism of deficient STAT3 activation during RSV infection of infant-derived cells. It also remains to be determined if STAT3 activation pathways are similarly deficient in airway epithelial cells from older individuals, the other population highly susceptible to severe RSV disease.

## Concluding remarks and perspectives

Largely attributed to naive immunity and narrower airway lumens, infants are more likely than adults to develop severe RSV infections. Zhao et al. importantly illustrate cell-autonomous STAT3 hyporesponsiveness of pediatric respiratory epithelial cells that increases apoptosis and shedding of RSV-infected cells, thus facilitating virus spread (12). Countering viral susceptibility by enhancing STAT3 activity or downstream effector pathways is not without risk, as STAT3 gain of function mutations trigger autoimmunity (23). Although RSV is a predominant cause of viral bronchiolitis in infants, human parainfluenza viruses, human metapneumoviruses, and rhinoviruses are also more severe in very young children. It will be important to determine whether the extent of infection by these viruses is also greater in pediatric-derived cultures and whether STAT3 status affects other respiratory viruses or is specific to RSV. In view of the effect of severe early childhood respiratory viral infections to increase asthma risk (24), the role of epithelial STAT3 activation status in all types of respiratory viral infections has important global health implications. Whether the STAT3 pathway can be safely exploited for effective antiviral therapy remains a future challenge.

## Version 1 11/01/2024

Electronic publication
